# Optimizing Field Emission Properties of the Hybrid Structures of Graphene Stretched on Patterned and Size-controllable SiNWs

**DOI:** 10.1038/srep15035

**Published:** 2015-10-19

**Authors:** Shasha Lv, Zhengcao Li, Jiecui Liao, Guojing Wang, Mingyang Li, Wei Miao

**Affiliations:** 1 State Key Laboratory of New Ceramics and Fine Processing, School of Materials Science and Engineering, Tsinghua University, Beijing 100084, China; 2 Key Laboratory of Advanced Materials (MOE), School of Materials Science and Engineering, Tsinghua University, Beijing 100084, China; 3 Department of Engineering Physics, Tsinghua University, Beijing 100084, China

## Abstract

Graphene is one of the ideal nanomaterials to be paired with silicon, and their complementary properties can be exploited in field emission (FE) devices. We reported an efficient way to produce and adjust the dimension of uniform protrusions within graphene. First, a multistep template replication process was utilized to fabricate highly periodic and well-aligned silicon nanowires (SiNWs) of different diameters (400, 500 and 600 nm). Then, large-scale and uniform graphene, fabricated by chemical vapor deposition (CVD), was transferred onto these size-controlled SiNWs to obtain the nanoscale and uniform undulations. As compared to the nanowires alone, the hybrid structures lead to higher FE performance due to electron conductivity enhancement, high-density emmison protrusions and band bending. These hybrid SiNWs/graphene structures could provide a promising class of field emission cathodes.

The silicon-based field emission devices are potential electron sources^1^,^2^, which can be applied in high energy accelerators, electron microscopes, X-ray sources, field emission (FE) light sources and microwave amplifiers. Addition of a secondary material whose properties complement the existing nanostructure could yield a combined characteristic. For this reason, various efforts have been made to fabricate silicon-based heterojunction nanostructures as field emitters^3^. In particular, graphene/silicon heterostructures are found to exhibit a high current density at a comparatively low electric field, enhancing the efficiency of FE devices^4^.

It is well known that graphene has a large surface area, flexible structure, sharp edges, unique geometry and favourable electric properties^5^,^6^,^7^. Therefore, it is recognized as an attractive material for its potential applications in multifunctional microelectronics devices^8^. Generally speaking, graphene can be synthesized by CVD^9^, plasma-enhanced CVD^10^, chemical exfoliation method^11^, etc. However, field emission efficiency from flat sheet structure of graphene is low, because electrons emit only from the sharp edges due to the decrease of work function of sheet edges^12^. Thus, graphene supported and stretched on the SiNWs may offer uniform and nanometer-scale protrusions, which could enhance FE properties and improve performance of silicon based devices^13^.

In general, field emission properties of nanostructured emitters depend on many factors^14^, including emitter dimension, aspect ratio (length/diameter)^15^, inter-distance^16^ and work function^17^. However, the issue regarding the uniformity and dimensional control of graphene protrusions by supporting on SiNWs has not been sufficiently addressed so far. In this context, we reported an effective and simple approach towards full control over the diameter of uniform SiNWs, then adjusted the protrusions of large-scale graphene sheet deposited on them. Furthermore, FE properties from aligned SiNWs and SiNWs/G heterostructures were systematically clarified.

## Results and Discussion

**Figure 1:**
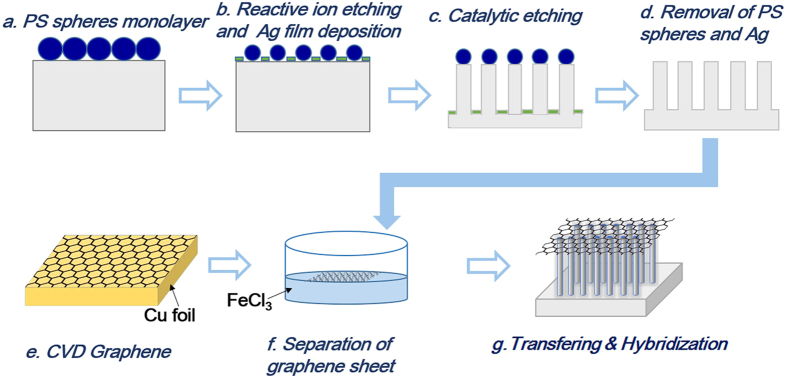
The procedures for fabrication of SiNWs/Graphene heterostructures.

**Figure 2:**
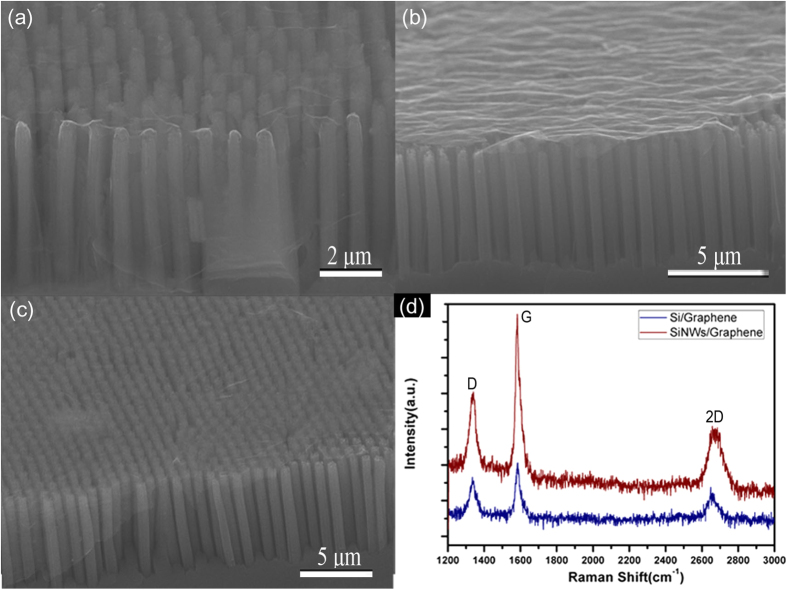
SEM images of graphene supported on SiNWs with diameter of (a) 400 nm, (b) 500 nm. (c) 600 nm, (d) corresponding Raman pattern of 400 nm SiNWs/Graphene.

Figure 1 schematically depicts the processes to fabricate the highly uniformed SiNWs/G heterostructures, and the morphology of the obtained SiNWs/G was characterized by scanning electron microscopy(SEM). Figure 2a–c show the cross-sectional images of the graphene supported by well-aligned and uniformly distributed SiNWs with a diameter of 400 nm, 500 nm and 600 nm, respectively, whereas their length is all ~5 μm. These SiNWs exhibit good monocrystallization, uniform alignment and long-range hexagonal periodicity. The mean diameter of the nanowires is controlled by the RIE etching time. Without using the PMMA film, we can directly transfer the graphene onto the SiNWs and prevent graphene from collapsing or breaking, which avoids the introduction of contamination to samples. The sheets conformally covered the surface of nanowires, leading to the formation of similarly shaped and high-density protrusions in the graphene.

The SiNWs/G hybrids and graphene on the Si substrate were investigated through Raman spectroscopy (Fig. 2d). The graphene related peaks are ~1384, 1582 and 2693 cm^−1^, corresponding to the D, G and 2G modes. The D band arises from backscattering of phonons by edges and defects like corrugation, twisting and edges. The G band is associated with doubly degenerate E_2g_ phonon modes of the *sp*^2^ hybridized carbon network of graphite. A symmetric 2D peak originates from a second-order process, indicating the graphene layers^18^,^19^,^20^. In SiNWs/G heterostructures, these three bands present higher intensity peaks compared with graphene transferred on silicon substrate. The intensity ratio of the D-to-G peaks can be applied to indicate the disorder degree of graphene. The *I*_*D*_*/I*_*G*_ value of SiNWs/G is 0.69, lower than that (0.90) of graphene on silicon. These signals confirm that good quality graphene was maintained after transfering to SiNWs. Additionally, the intensity ratio of G to 2D bands (*I*_*G*_*/I*_*2D*_) is 1.58, which indicates it’s not the monolayered graphene.

**Figure 3:**
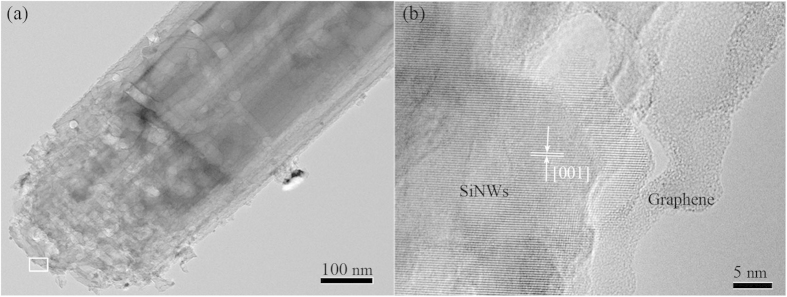
TEM images of (a) SiNWs/Graphene heterostructures under a low magnification, (b) the layers of coated graphene, the corresponding HRTEM image of the (c) tip and (d) side region in (a).

The TEM and HRTEM were performed to study the formation of protruded graphene layers on top of the SiNWs. From Fig. 3a, it is seen that the rough top surface of SiNWs leave many small and sharp tips, thus creating more local protrusions within the graphene sheets and revealing the existing emission sites. Figure 3b shows the HRTEM image of the interface regions of square section in Fig. 3a, where SiNWs with a [001] orientation are covered by graphene nanosheets.

**Figure 4:**
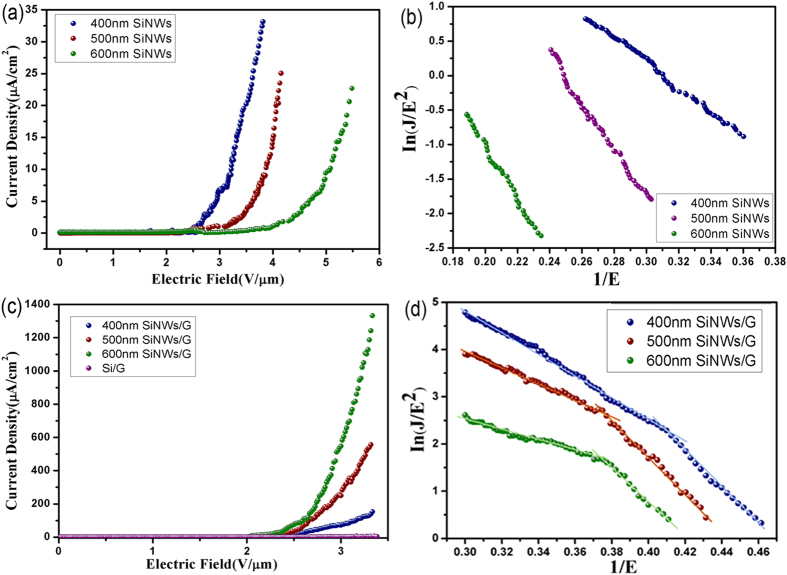
(**a**) *J-E* curves and (**b**) F-N plots of SiNWs with diameter of 400 nm, 500 nm and 600 nm; (**c**) *J-E* curves and (**d**) F-N plots of graphene supported on silicon substrate and SiNWs with diameter of 400 nm, 500 nm and 600 nm.

Field electron emission is a quantum tunnelling phenomenon where electrons are emitted from a solid surface due to the effect of a strong electrostatic field. The field emission characteristics of SiNWs with different diameters (400 nm, 500 nm, 600 nm) and their corresponding heterostructures with graphene are shown in Fig. 4. Also, bare graphene sheets transferred on silicon substrate were tested for comparison, as displayed in Fig. 4c. The Fowler–Nordheim (F–N) formula is utilized to quantitatively describe the field emission process, and the current density (*J*) produced by a given electric field (*E*) is described as the following equation^21^:


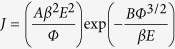


Where *A* and *B* are constants, *Φ* is the work function of the emitting materials, *β* is field enhancement factor. Usually, *β* was calculated from the slope of F–N plot^22^ according to the equation *k* *=* *−BΦ*^*1.5*^*d/β*. Here, *Φ* is 4.85 eV for n-Si (100) and 4.53 eV for graphene^23^ and *d* is the anode–cathode distance. Usually, the lower work function and larger tip radius of curvature contribute to the higher current density. The F–N plots corresponding to the samples under investigation were depicted in Fig. 4b,d. The enhancement factors were estimated from their corresponding F–N plot. For SiNWs of 400 nm, 500 nm and 600 nm, the turn-on field was 3.35, 4.27, 5.20 V/μm, and *β* was found to be 3727, 2142, 1875. The field enhancement factor can be estimated by *β* ∝ aspect ratio, thus SiNWs of 400 nm have a higher field enhancement factor than the other samples. It appears that electric field shielding effects from closely packed nanowires adversely affect their field emission characteristics. By reducing the diameter of nanowires and increasing the space between the SiNWs, field shielding effects can be efficiently reduced, and the FE performance was enhanced.

For SiNWs/G, the turn-on field values with diameters of 400, 500 and 600 nm are 2.59, 2.33, and 2.01 V/μm, respectively. The slopes of F–N plot of SiNWs/G (Fig. 4d) in the low field and high field are obviously different. The enhancement factors in the high electric field region, were found to be 4044, 4369, 6513, for graphene supported on SiNWs of 400 nm, 500 nm, 600 nm, respectively. The downbending F-N plot is usually observed for various nanoemitters with increase of the electric field^24^,^25^. In those cases, the field emission gradually emerges into a current saturation regime as field increases, due to two main factors. For one thing, the applied high electric field heats graphene surfaces, decreases its work function, and leads to an increase of the emission current^26^, for another, as the electric field increases, the number of emission sites increases, which changes the overall characteristic of the emitters.

**Figure 5:**
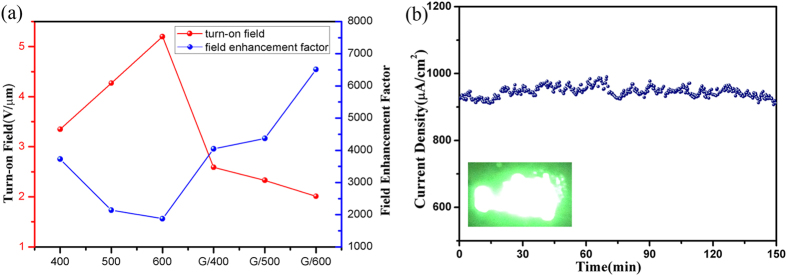
(**a**) Variation of turn-on field and field enhancement factor with different samples. (**b**) FE stability of 600 nm SiNWs/Graphene.

The field emission parameters, such as the turn-on field and enhancement factor of the different samples, are summarised in Table 1 and Fig. 5a. It shows that SiNWs/G have lower turn-on field and higher *β* values, suggesting that a combination of graphene and SiNWs can effectively improve the FE properties. The 600 nm SiNWs tailored graphene composite displayed the best field emission with a threshold electric field of only 3.22 V/μm at 1 mA/cm^2^ emission current. Long-term stability of field emission is aslo a critical issue for continuous operation. To check the robustness of the graphene on 600 nm SiNWs, the field emission stability of G/SiNWs was recorded at a fixed electric field of 3.15 V/μm for 3 h. It shows an average emission current density of ~932 μA/cm^2^, without showing any sign of degradation (Fig. 5b). Therefore, high surface area and large thermal conductivity of graphene impart excellent stability to the emission current.

**Figure 6:**
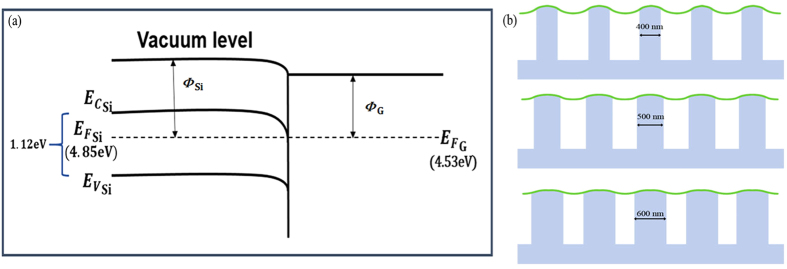
(**a**) Schematic energy band diagram of the SiNWs/Graphene interface. (**b**) The schematic model of graphene supported on SiNWs with different diameters.

The whole process of electron field emission consists of two transport steps: electrons transfer from SiNWs to graphene, followed by electrons tunnelling through the barrier from the graphene to vacuum. Due to band bending at the graphene–nanowire tip junction^27^,^28^,^29^, an electron traveling through the SiNWs falls in the downhill potential and thus can easily be transferred from nanowires to graphene, for the ohmic contact through the semiconductor NWs and metallic graphene substrate, as schematically shown in Fig. 6a. Therefore, the junction can level the dissimilar work function, and enhance the FE performance.

Another point to be explained is the *β* value. When two different sorts of nanostructures are combined, the hybrids will serve as a two-stage field emitter. The field enhancement factor of the cascade emitter can be presented as^30^


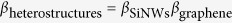


Where *β*_SiNWs_ and *β*_graphene_ are field enhancement factor of the SiNWs and the graphene, respectively. The presence of graphene edges is a key factor responsible for the excellent FE proprerty. In addition to the uniform undulation resulting from the well-aligned SiNWs, the rough top surface of SiNWs tips also lead to nanoscale protrusions. Each protrusion serves as one field emitter. Therefore, the value of *β*_graphene_ indicates distributed undulations and protrusions in the graphene, and the *β* value of heterostructures is usually lager than the *β*_SiNWs_. Graphene coverage decreases the electric field penetration of the hybrid SiNWs, therefore, the *β*_SiNWs_ is decreased when compared to bare SiNWs. From the above results, we can see that the *β* value is determined not only by the geometrical characteristics of emitters, but also by the spatial density.

The schematic model of graphene supported on SiNWs with different diameters(400 nm, 500 nm and 600 nm) is shown in Fig. 6b. The turn-on field difference of the hybrid structure with different diameters is relatively small. Furthermore, the large-scale graphene on SiNWs with a diameter of 600 nm has lowest turn-on field and highest *β* values, because this hybrid offers more contact points and protrusions between SiNWs and graphene, and leads electrons transfering more easily. The presence of graphene protrusions is a key factor responsible for the excellent field emission properties of hybrids. As a consequence of graphene interlinking^31^, nanowire tips are capped with graphene to facilitate electron emission via band bending. It is expected that the advantages of SiNWs/graphene based nanomaterials have the high aspect ratio, stable structure, and outstanding electrical properties^32^,^33^,^34^,^35^,^36^,^37^,^38^,^39^,^40^,^41^.

In conclusion, we report an effective approach to control the uniform SiNWs with precisely tunable diameters and proximity, relative to the PS sphere size and catalyzed etching time. Then, large scale, uniform graphene was transferred onto well-aligned SiNWs. On one hand, SiNWs provide the graphene sheet with uniform and nanometer-scale protrusions. On the other hand, the emitter density and inter-protrusion distance can be manipulated by the size of the nanowires. For bare SiNWs, the FE properties were significantly increased as the SiNWs diameter decreased and proximity increased. Conversely, the hybrid structure of stretched graphene on the tips of the SiNWs exhibited lower turn-on electric field and higher *β* value as the SiNWs diameter increased. The graphene supported on the SiNWs of 600 nm in diameter have an ultra-low turn-on field (2.01 V/μm) and threshold electric field (3.22 V/μm), and they are observed to have predominant FE stability. The highly efficient and stable field emission performance is because of electron conductivity enhancement, high-density emission spots and band bending. These high-density protrusions localize and enhance the local electric field. Furthermore, the band bending at the SiNWs–graphene junctions level their dissimilar work functions, allowing for easy electron transfer from the SiNWs to the graphene. Therefore, the graphene protrusions, with a controlled period in the graphene sheet, may act as excellent field emitters.

## Methods

### Fabrication procedure of the size-controllable SiNWs

The n-type (100) Si pieces were degreased by successive ultrasonic cleaning in acetone, ethanol, and deionized water for 15 min each, and then immersed into boiling acid solution (4:1 (v/v) H_2_SO_4_/H_2_O_2_) for 1 h. Subsequently, monodispered PS nanosphere template of 960 nm in diameter was prepared by the air-water interface self-assembly method. Next, pretreated silicon substrates were placed and pushed to the below side of the compact PS spheres, and the monolayer of the PS nanosphere template could be transferred onto the Si substrate upon completion of the water evaporation.

The diameter of PS spheres was reduced by applying a different time of reactive ion etching (RIE) with an O_2_ flow rate of 40 sccm, basic pressure of 2 Pa, and a radio frequency power of 30 W. After that, the Ag film was deposited onto the Si substrate by magnetron sputtering, forming a porous Ag film as catalyst. Subsequently, the Ag film coated Si substrate was etched in a mixed solution of HF and H_2_O_2_ at 30 °C for 15 min, where the concentrations of HF and H_2_O_2_ were 4.8 and 0.3 M, respectively. Finally, the PS sphere templates were removed by ultrasonication in toluene, and the retained Ag film was dissolved with HNO_3_^42^.

### Preparation and transfer of graphene

The graphene used in our work was originally grown on copper foil by the CVD method^43^. For the graphene transfer, the copper was completely dissolved in a ferric chloride solution and the graphene floated on the surface of the etchant. After being cleaned three times with deionized water, the graphene film was manually lifted out from water by the SiNWs coated silicon substrate.

### Characterization and FE measurement

The morphology and microstructure of the heterostructures were observed by field emission scanning electron microscopy (FE-SEM, JEOL-JSM 7001F, Tokyo, Japan), and transmission electron microscopy (TEM, JEOL-JSM 2011). Raman-scattering spectra were collected using an HORIBA Jobin Yvon HR800 Spectrometer (laser of 562 nm). For testing the field emission properties, the samples were fixed onto the cathode, and the phosphor was deposited on a transparent conductive material (indium-tin-oxide) to serve as the anode electrode in the vacuum system. The distance between the cathode and anode was kept to ~300 μm. A voltage was applied between the anode and cathode to supply an electric field. The emission current was measured by increasing a voltage.

## Additional Information

**How to cite this article**: Lv, S. *et al.* Optimizing Field Emission Properties of the Hybrid Structures of Graphene Stretched on Patterned and Size-controllable SiNWs. *Sci. Rep.* **5**, 15035; doi: 10.1038/srep15035 (2015).

## Figures and Tables

**Table 1:** Key parameters of SiNWs and SiNWs/Graphene field emitters in this work.

Samples	turn-on field (V/μm)	*β*
400 SiNWs	3.35	3727
500 SiNWs	4.27	2142
600 SiNWs	5.20	1875
G/400 SiNWs	2.59	4044
G/500 SiNWs	2.33	4369
G/600 SiNWs	2.01	6513
